# The Role of a Betaretrovirus in Human Breast Cancer: Enveloping a Conundrum

**DOI:** 10.3390/v14112342

**Published:** 2022-10-25

**Authors:** Walter H. Gunzburg, Brian Salmons

**Affiliations:** 1 Department of Pathobiology, Institute of Virology, University of Veterinary Medicine, A-1210 Vienna, Austria; 2 Austrianova, The Gemini, Science Park 2, Singapore 117610, Singapore

**Keywords:** mouse mammary tumor virus, human mammary tumour virus, human betaretrovirus, envelope, receptor, tumorigenesis, breast cancer

## Abstract

Most of the evidence that a human betaretrovirus (HBRV/HMTV) highly related to mouse mammary tumour virus (MMTV) has an etiological role in breast cancer has been summarized in a recent comprehensive Special Issue of “Viruses” entitled “Human Betaretrovirus (HBRV) and Related Diseases”. Shortly after publication of this special issue, a detailed analysis of aligned env sequences was published and concluded that (i) MMTV and HBRV/HMTV cannot be distinguished on the basis of aligned env sequences and (ii) more sequence data covering the full-length env or HBRV/HMTV genomes from multiple isolates is needed. Although productive infection of human cells by MMTV (and presumably HBRV/HMTV) has been shown, it is imperative that the receptor(s) enabling HBRV/HMTV to infect human cells are defined. Moreover, there is currently no compelling data for common integration sites, in contrast to MMTV induced mammary tumorigenesis in mice, suggesting that other mechanisms of tumorigenesis are associated with HBRV/HMTV infection. These issues need to be resolved before a clear link between MMTV/HBRV/HMTV and human breast cancer can be concluded.

We read with great interest the recent Special Issue “Human Betaretrovirus (HBRV) and Related Diseases” that was published in “Viruses” and the various lines of evidence for an etiological role of the virus in human disease, fulfilling many of the Bradford Hill criteria [1]. Shortly after the publication of this comprehensive issue, a pertinent publication by Ahmad and colleagues [2] appeared in which they reported extensive analyses of envelope (env) sequences from mouse mammary tumour virus (MMTV) isolates and compared them with the available env sequences from the human betaretrovirus [3], referred to as human mammary tumour virus (HMTV) in the Ahmad publication [2]. The in depth analysis concluded that MMTV and HBRV/HMTV could not be distinguished on the basis of aligned env sequences. The authors conclude that HBRV/HMTV cannot be considered a separate species from MMTV until more sequence data covering the full-length env or HBRV/HMTV genomes from multiple isolates becomes available [2]. The current data, however, would seem to support a proposed zoonotic transmission of MMTV [4].

The authors point out that one reason for the inability to distinguish MMTV from HMTV could be that most groups have sequenced only a ~700 bp region of HMTV (nt 7240–7908) whereas the whole env coding sequence is over 2000 bp long. Nevertheless, this 700 bp region is within the SU region of Env that has been reported for MMTV to contain the domain that binds to the viral cellular receptor, mTfR1. However, binding to human TfR1 (hTfR1) alone is not sufficient to support the successful entry of MMTV into human cells [5,6]. The authors thus propose that sequence variation outside the receptor binding domain may reflect either a different step of viral entry or a different mode of entry [2]. We strongly agree with their conclusion that more work needs to be done to sequence all parts of the viral genome, including the cloning of further full length HBRV/HMTV integrated proviral genomes, preferably from DNA isolated from patients with breast cancer.

Clearly, there is also a need to identify other receptors that can mediate infection of human cells by MMTV. Previous publications have shown that MMTV can infect human cells by transfer of cell free supernatant containing virus [7] and this infection is productive i.e., leads to multiple rounds of infection of human cells [8]. Indeed, persistently infected human Hs578T cells produced infectious virions capable of infecting naïve human breast cells in culture. This infectivity can also be blocked by neutralizing antibodies raised against MMTV produced by mouse cells, demonstrating that virus particles released by the persistently infected Hs578T cells were related antigenically to the virus produced from murine cells. Moreover, in one study using human cells infected in cell culture, hundreds of unique integration sites were mapped from the infected human cells and each integrated provirus shown to have the characteristic 6 bp duplication of host sequence at the site of integration [9,10]. Other evidence for one or more receptor(s) other than TfR1 mediating the infection of non-heterologous cells includes the finding that although the Crandell feline kidney cell line (CrFK) is well known to be susceptible to MMTV [11,12], the transfer of the feline TfR1 to other cells does not make cells susceptible to MMTV infection [13]. Thus, feline cells must express another molecule, different from TfR1, that mediates virus entry into cells.

To date, there is no evidence to support that HBRV/HMTV causes human breast cancer via common insertion sites (enhancer insertion), leading to inappropriate expression of cellular proto-oncogenes, followed by clonal expansion of these infected cells. This is, however, the currently accepted mechanism by which MMTV causes mammary tumors in mice [14]. Moreover, although there are numerous publications showing infection of human cells by MMTV by mapping integration sites in the human genome, both in cell culture [7,8,9,10], as well as in DNA from in vitro infected cells [15] as well as from patients with primary biliary cholangitis (PBC) [16], common integration sites have not yet been shown in human breast cancer [2,16,17]. Also, it should be noted that the recent studies in patients with PBC (an autoimmune liver disease) support a recessive loss of gene function of tumor suppressor genes such as PTEN and RANBP2 via insertional mutagenesis or aberrant splicing as a mode of action as opposed to the dominant MMTV-enhancer driven activation of proto-oncogenes [14]. These observations have led to other mechanisms being proposed by which HBRV/HMTV could transform breast epithelial cells other than by enhancer insertion and some of these mechanisms have precedents in the transformation of human cells by other retroviruses [3,17,18,19]. These mechanisms may also involve interactions with other viruses that have been linked to human breast cancer [20,21]. Clearly, the elucidation of the functional receptor(s) mediating infection of human cells as well as the actual mechanism(s) by which the virus contributes to the genesis of breast cancer remain the major stumbling block(s) before a clear link between MMTV/HBRV/HMTV and human breast cancer can be concluded.
